# fMRI evidence for areas that process surface gloss in the human visual cortex

**DOI:** 10.1016/j.visres.2014.11.012

**Published:** 2015-04

**Authors:** Hua-Chun Sun, Hiroshi Ban, Massimiliano Di Luca, Andrew E. Welchman

**Affiliations:** a School of Psychology, University of Birmingham, UK; b Center for Information and Neural Networks, National Institute of Information and Communications Technology, Osaka, Japan; c Graduate School of Frontier Biosciences, Osaka University, Osaka, Japan; d Japan Society for the Promotion of Science, Tokyo, Japan; e Department of Psychology, University of Cambridge, UK

**Keywords:** Surface gloss, Material perception, Posterior fusiform sulcus, V3B/KO, fMRI

## Abstract

Surface gloss is an important cue to the material properties of objects. Recent progress in the study of macaque’s brain has increased our understating of the areas involved in processing information about gloss, however the homologies with the human brain are not yet fully understood. Here we used human functional magnetic resonance imaging (fMRI) measurements to localize brain areas preferentially responding to glossy objects. We measured cortical activity for thirty-two rendered three-dimensional objects that had either Lambertian or specular surface properties. To control for differences in image structure, we overlaid a grid on the images and scrambled its cells. We found activations related to gloss in the posterior fusiform sulcus (pFs) and in area V3B/KO. Subsequent analysis with Granger causality mapping indicated that V3B/KO processes gloss information differently than pFs. Our results identify a small network of mid-level visual areas whose activity may be important in supporting the perception of surface gloss.

## Highlights

• Glossiness information is mainly processed along ventral visual pathway.
• The posterior fusiform sulcus (pFs) is especially selective to surface gloss.
• V3B/KO responds to gloss, but differentially from the pFs.

## 1 Introduction

Surface gloss provides an important cue to an object’s physical material and its microstructure (Nishio, Goda, & Komatsu, 2012). From a perceptual perspective, it has particularly intriguing properties because there are cases where glossiness is specified only by small image areas containing highlights (Beck, 1972). Unlike other aspects of material, a slight change in an object (e.g. minor change of material or smoothness) can cause huge differences in the perceptual impression of gloss (Fleming, 2012). While a number of image cues have been proposed to modulate gloss perception, it is an open challenge to understand how this information is processed to infer surface material.

Psychophysical studies suggest that the brain uses a variety of visual signals to estimate gloss. For instance, low-level factors such as the image luminance histogram skew can bias perceived gloss and cause perceptual aftereffects (Gegenfurtner, Baumgartner, & Wiebel, 2013; Motoyoshi, Nishida, Sharan, & Adelson, 2007). Mid-level factors such as specular reflections (Nishio et al., 2012; Norman, Todd, & Orban, 2004; Okazawa, Goda, & Komatsu, 2012; Okazawa, Koida, & Komatsu, 2011; Wendt, Faul, Ekroll, & Mausfeld, 2010) and surface relief (Anderson, Marlow, & Kim, 2012; Marlow & Anderson, 2013; Marlow, Kim, & Anderson, 2012; Wijntjes & Pont, 2010) also influence the impression of gloss. Highlights play a particularly important role in affecting judgments of material, and this can relate to their position and orientation (Anderson & Kim, 2009; Anderson, Kim, & Marlow, 2011; Kim & Anderson, 2010; Kim, Marlow, & Anderson, 2011; Marlow, Kim, & Anderson, 2011), their colour (Nishida, Motoyoshi, & Maruya, 2011; Wendt et al., 2010), and their binocular disparity (Kerrigan & Adams, 2013; Muryy, Fleming, & Welchman, 2012; Wendt, Faul, & Mausfeld, 2008; Wendt et al., 2010). Here we chose to investigate how manipulating surface appearance through highlights gives rise to changes in brain activity. In particular, we use fMRI to identify the cortical regions that respond preferentially to visual gloss depicted by highlights.

Recent studies have suggested candidate areas in macaque brain that may play an important role in processing gloss (Nishio et al., 2012; Okazawa et al., 2012). For instance, specular objects elicited more fMRI activation along the ventral visual pathway, from V1, V2, V3, V4 to inferior temporal (IT) cortex compared to matte objects and phase-scrambled images of the objects (Okazawa et al., 2012). Single-unit recordings from the superior temporal sulcus (STS) within IT cortex identified neurons that were selective for gloss uninfluenced by changes in the 3D structure of the viewed object or by changes to the illumination (Nishio et al., 2012). Further, these gloss-selective responses reflect combinations of reflectance parameters that align to the perceptual dimensions guide judgments of surface properties (Nishio, Shimokawa, Goda, & Komatsu, 2014). These results from the macaque indicate that specular reflectance properties are likely to be encoded in ventral visual areas.

Despite this recent progress in the macaque model, we still have rather little insight into how the human brain processes gloss. Human brain imaging work examining the (more general) representation of material properties (e.g. wood vs. metal) implicated a role of ventral visual areas, especially in fusiform gyrus (FG), inferior occipital gyrus (IOG) and collateral sulcus (CoS) (Cant, Arnott, & Goodale, 2009; Cant & Goodale, 2007; Hiramatsu, Goda, & Komatsu, 2011). This work employed stimulus changes in multiple image dimensions (e.g. colour, texture and gloss), meaning that activity related to gloss *per se* could not be determined. It is likely to be an important distinction as tests of a neuropsychological patient who had deficits in colour and texture discrimination showed that they were unimpaired on gloss judgments (Kentridge, Thomson, & Heywood, 2012). This suggests that the cortical processing of gloss is (at least partially) independent from the processing of other material properties. Recently, Wada and colleagues (Wada, Sakano, & Ando, 2014) reported that fMRI activity related to surface gloss is evident in V2, V3, V4, VO-1, VO-2, CoS, LO-1 and V3A/B. In particular, they contrasted glossy and matte objects under bright and dim illumination to exclude the confounding of luminance. Here we use the different approach of perturbing global image arrangement while preserving local image features to target mechanisms of the global synthesis of image cues when judging gloss. It is also different from Okazawa et al. (2012) who contrasted glossy objects with phase-scrambled versions of these objects. We presented observers with stimuli from four experimental conditions: Glossy, Scrambled Glossy, Matte and Scrambled Matte. Thereby we sought to discriminate Gloss vs. Matte renderings of objects while dissociating the role played by local image features.

## 2 Methods

### 2.1 Participants

Fifteen participants who had normal or corrected-to-normal vision were recruited for the experiment. Two were authors (H.-C. S. and H. B.) and the remainder were naïve participants. All were screened for normal stereoacuity and MRI safety before being invited to participate. All participants had previously participated in other fMRI studies in which fMRI localiser data (see ‘ROI definition’) and a T1-weighted anatomical scans (see ‘MRI data acquisition’) were acquired. The age range was 19–35 years old, and 13 of the 15 were male. All participants gave written informed consent before taking part in the experiment. The study was approved by the STEM Ethical Review Committee of the University of Birmingham. The work was carried out in accordance with The Code of Ethics of the World Medical Association (Declaration of Helsinki). After completing the experiment, non-lab member participants received monetary compensation.

### 2.2 Apparatus and stimuli

#### 2.2.1 Stimuli

**Fig. 1:**
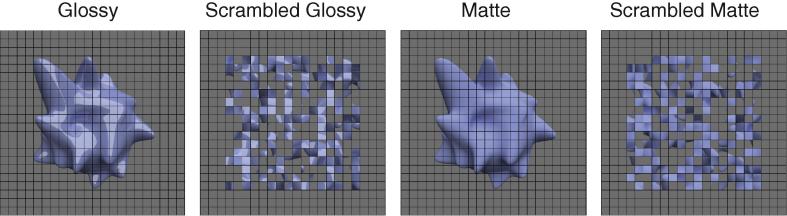
The four experiment conditions (Glossy, Scrambled Glossy, Matte and Scrambled Matte) rendered on an example object. Glossy components were shown in the Glossy condition while only the diffuse components (Lambertian reflectance function) were shown in Matte condition. In the scrambled conditions, a 22 × 22 grid was superimposed over the images and then squares were randomly relocated within the grid.

The stimuli comprised 32 2-D renderings of 3-D objects generated in Blender 2.67a (The Blender project: http://www.blender.org/). The objects were spheres and tori whose surfaces were perturbed by random radial distortions to produce slightly irregular shapes. The diameter of the stimuli was 12° on average and they were presented on a mid-gray background. We illuminated the objects using a square light source located front and above the objects. We chose this simple light source to be able to increase the influence of our scrambling manipulation. We created versions of the stimuli for each object that made up the four conditions of the experiment: Glossy, Scrambled Glossy, Matte and Scrambled Matte (Fig. 1). In the Glossy condition, objects were rendered using a mixed shader with 90% diffuse and 10% glossy components. We rendered objects in the Matte condition by setting the reflectance function to Lambertian (100% diffuse component). We controlled the luminance of the stimuli so that the mean luminance of the stimuli was 60.54 cd/m^2^ and the absolute maximum was 103.92 cd/m^2^ which corresponded to 57.55% and 98.78% of the display maximum luminance, respectively. All the objects were rendered without background then we set background colour to gray before further manipulations as described below.

To produce spatial scrambling, we superimposed a 22 × 22 1-pixel black grid over the images and then randomly relocated squares (0.55° of side) within the grid (Kourtzi & Kanwisher, 2000; Malach et al., 1995). This approach differs from phase scrambling (Okazawa et al., 2012) as blur, contrast, and luminance are only marginally affected. Moreover, the mosaic spatial scrambling approach we used interrupts object shape, shading, and specular highlights while all the local information (e.g., luminance, contrast, luminance histogram skew) is unchanged. Previous work indicates that highlight congruence with surface geometry and shading is crucial for perceived glossiness (Anderson & Kim, 2009; Kim et al., 2011; Marlow et al., 2011). Thus our stimuli strongly attenuate the impression of gloss by disrupting the relationship between highlights and global object structure.

**Fig. 2:**
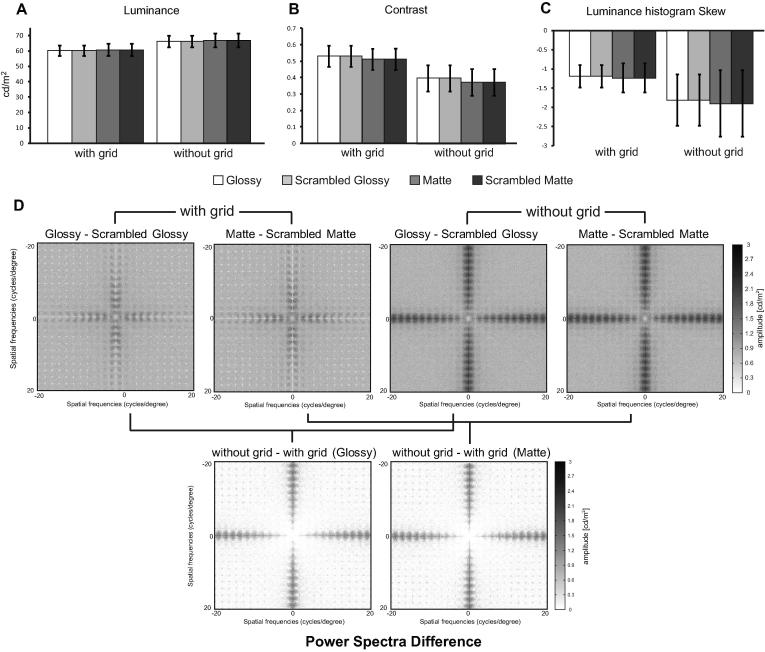
Image statistics of (A) pixelwise luminance, (B) contrast, (C) histogram skew, and (D) difference in power spectra across the 32 images with and without the superimposed grid. Luminance was calculated by averaging the mean luminance of all pixels in each image then averaging across images. Contrast was calculated with pixelwise luminance’s standard deviation divided by its mean for each image, averaged across images. Skew was calculated as the third standardized momentum of the luminance histogram of each image, averaged across images (Motoyoshi et al., 2007). The absolute difference in power spectra was calculated for each image pair and then averaged across images.

Note that the superimposed grid was presented for both intact and scrambled versions of the stimuli. This greatly attenuates the amount of additional edge information that results from the spatial scrambling manipulation. Formally, we assessed differences in image structure by computing possible image cues that might drive the fMRI response. In particular, we found that the image statistics of mean luminance, luminance root-mean-square contrast, and luminance histogram skew were matched across the four conditions (Fig. 2) indicating that there was more variation within the same class of stimuli than there was between classes. This is trivial for the scrambled versions of the stimuli (they must have the same values of skew, contrast and luminance, by definition), however, it is important that matte and glossy stimuli were well matched. In such a case, although the addition of a grid affects all these measures, it did not create any consistent difference across the four conditions, thus the interpretation of the results should not be affected. Furthermore, the power spectra of the stimuli in the different conditions (Fig. 2D) indicate that the grid is effective in equalizing the spatial frequency content of the images, particularly when contrasted with scrambled images without a superimposed grid. The grid adds high frequency components to intact images creating a pattern that is very similar to the one due to the scrambling procedure. In this way, frequency spectra are made more similar across conditions.

#### 2.2.2 Apparatus

Stimulus presentation was controlled using MATLAB (Mathworks Inc.) and Psychtoolbox (Brainard, 1997; Pelli, 1997). The stimuli were back projected from a JVC DILA SX21 projector onto a translucent screen inside the bore of the magnet. Participants viewed the stimuli binocularly via a mirror fixed on the head coil with a viewing distance of 64 cm. Luminance outputs were linearized and equated for the RGB channels separately with colorimeter measurements. A five-button optic fiber button box was provided to allow responses during the 1-back task.

#### 2.2.3 MRI data acquisition

A 3-Tesla Philips scanner and a 32-channel phase-array head coil were used to obtain all MRI images at the Birmingham University Imaging Centre (BUIC). Functional whole brain scans with echo-planar imaging (EPI) sequence (32 slices, TR 2000 ms, TE 35 ms, voxel size 2.5 × 2.5 × 3 mm, flip angle 80°, matrix size 96 × 94) were obtained for each participant. The EPI images were acquired in an ascending interleaved order for all participants. T1-weighted high-resolution anatomical scans (sagittal 175 slices, TR 8.4 ms, TE 3.8 ms, flip angle 8 deg, voxel size: 1 mm^3^) were obtained from previous studies.

### 2.3 Design and procedure

**Fig. 3:**
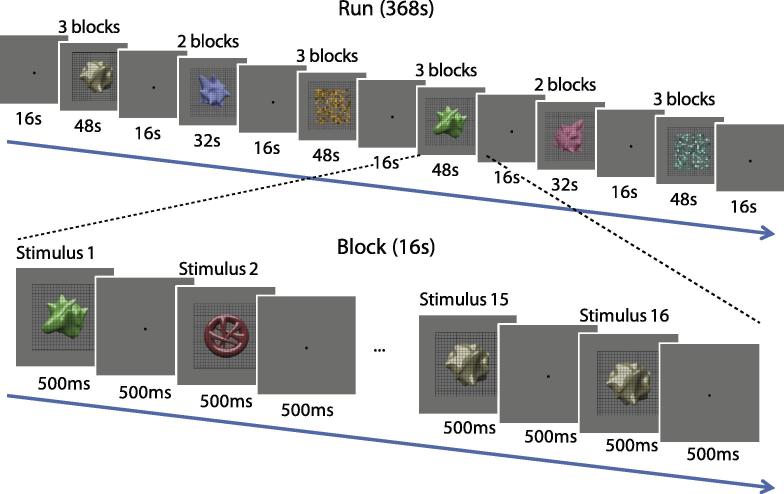
The fMRI procedure for one scan run. One each run there were 23 blocks (16 s each), including 7 fixation blocks and 16 experimental blocks. During each experimental block, stimuli were presented for 500 ms with 500 ms interstimulus interval (ISI). Participants were instructed to perform a 1-back matching task.

A block design was used. Each participant took part in 8–10 runs with 368 s length of each run in a 1.5 h session. Each run started with four dummy scans to prevent startup magnetization transients and it consisted of 16 experimental blocks each lasting 16 s. There were 4 block types (i.e., one for each condition), repeated four times in a run. During each block, eight objects were presented twice in a pseudo-random order. Stimuli were presented for 500 ms with 500 ms interstimulus interval (ISI). Participants were instructed to maintain fixation and perform a 1-back matching task, whereby they pressed a button if the same image was presented twice in a row. They were able to perform this task very well (mean *d*′ > 3). Five 16 s fixation blocks were interposed after the third, fifth, eighth, eleventh and thirteenth stimulus blocks to measure fMRI signal baseline. In addition, 16 s fixation blocks were interposed at the beginning and at the end of the scan, making a total of seven fixation blocks during one experimental run. An illustration of the scan procedure is provided in Fig. 3.

### 2.4 Data analysis

#### 2.4.1 Functional MRI data processing

BrainVoyager QX version 2.6 (Brain Innovation, Maastricht, The Netherlands) was used for MRI data processing. Each participant’s left/right cortical surfaces were reconstructed by segmenting gray and white matter, reconstructing the surfaces, inflating, cutting and then unfolding. All functional images were pre-processed with slice scan timing correction, 3D head motion correction, high-pass filtering (2 cycles per run cut-off) and linear trend removal. Functional images were co-registered with anatomical images and then transformed to Talairach coordinate space and aligned with each other. We computed the global signal variance of the blood oxygenation level dependent (BOLD) signal for each run using the whole-brain average of activity across volumes. If this exceeded 0.16% the scan run was excluded from further analysis to avoid the influence of scanner drifts, physiological noise or other artifacts (Junghöfer, Schupp, Stark, & Vaitl, 2005). On this basis, 17/146 runs across 15 participants were excluded from further analysis. A 3D Gaussian spatial smoothing kernel with 5 mm full-width-half-maximum (FWHM) was applied before analysing the data using a group-level random effects (RFX) general linear model (GLM).

#### 2.4.2 ROI definition

A total of 11 regions of interest (ROIs) were defined. For each participant V1, V2, V3d, V3A, V3v, V4 were drawn by visual inspection of the data obtained from a standard retinotopic mapping scan preceding the experiment (Preston, Li, Kourtzi, & Welchman, 2008). V3B/KO (kinetic occipital region), hMT+/V5 (human motion complex) and LO (lateral occipital region) were defined by additional functional localizers respectively in a separate session as in previous studies (Ban, Preston, Meeson, & Welchman, 2012; Dövencioğlu, Ban, Schofield, & Welchman, 2013; Murphy, Ban, & Welchman, 2013). For nine of the fifteen participants, V3B/KO and hMT+/V5 were defined according to Talairach coordinates ([*x*,*y*,*z*] = [42, −81, 6] for right V3B/KO; [−42, −81, 6] for left V3B/KO; [42, −62, 6] for right hMT+/V5; [−42, −66, 2] for left hMT+/V5) (Orban et al., 2003; Sunaert, van Hecke, Marchal, & Orban, 1999). LO and pFs were defined by a localizer scan for all participants in which intact object images and their spatially-scrambled versions were contrasted. pFs was identified as the more anterior portion of the activation map obtained from this contrast. The average mass centre of LO and pFs across the 15 participants were [39, −63, −8] and [31, −43, −14] for right and [−40, −67, −4] and [−38, −48, −14] for left hemisphere. The superior temporal sulcus (STS) was defined according to Talairach coordinates ([57, −45, 10] for right and [−57, −45, 10] for left in superior temporal sulcus) (Sunaert et al., 1999).

#### 2.4.3 Additional fMRI analysis

We computed percent signal change (PSC) by subtracting the BOLD signal baseline (the average signal in fixation blocks) from each experimental condition and then dividing by the baseline. In addition, voxels used in the PSC analysis were masked with the *t*-value maps obtained by contrasting all stimulus conditions vs. fixation blocks for each individual participant. PSCs were examined within independently identified ROI under each experiment condition. We then computed the difference in PSC between intact and scrambled versions of Glossy and Matte objects, which we term **Δ**PSC.

Finally, we used random effects Granger causality mapping (RFX GCM) to probe the information flow between ROIs. Granger causality uses temporal precedence to identify the direction of influence from a reference region to all other brain voxels (Roebroeck, Formisano, & Goebel, 2005). The GCMs for each participant were calculated first then they were combined together with a simple *t*-test (*t* > 0) and cluster-size thresholding (25 mm^2^).

## 3 Results

**Fig. 4:**
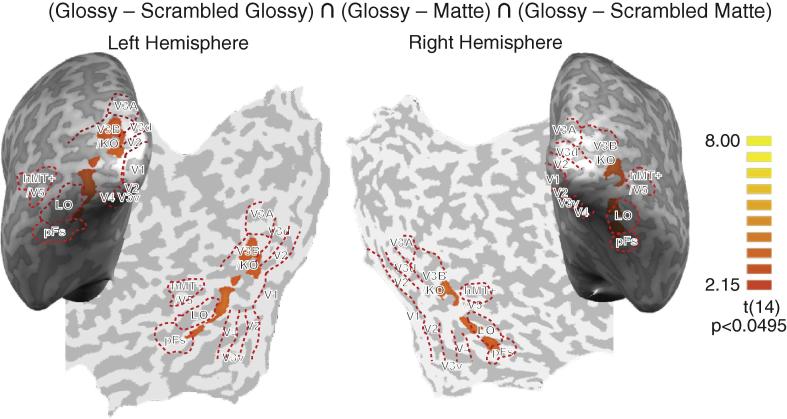
The result of conjunction analysis across the 15 participants. The Glossy condition was compared with the other three conditions. Significant conjunctions are presented on representative flat maps. Sulci are shown in dark gray and gyri are in light gray. The colour scale indicates *t*-values. The significance level was *p* < .05 with cluster-size thresholding 25 mm^2^. The orange areas represent activation in Glossy condition that is significantly higher than any of the other three conditions, respectively.

To identify brain areas that preferentially responded to glossy objects, we used a conjunction analysis to find voxels that were activated more strongly in Glossy condition than in any of the other three conditions across the 15 participants. In particular, Fig. 4 shows the results of a random-effects GLM with statistical significant voxels (*p* < .05) and cluster-size thresholding (25 mm^2^). The orange areas demark significantly higher activation in Glossy condition under the three contrasts, respectively: Glossy vs. Scrambled Glossy, Glossy vs. Matte, Glossy vs. Scrambled Matte. In general, these areas were distributed along ventral visual pathway in both hemispheres including the ventral occipitotemporal cortex. In addition, we found responses in the area around V3B/KO, which is traditionally thought to belong to the dorsal visual stream.

**Fig. 5:**
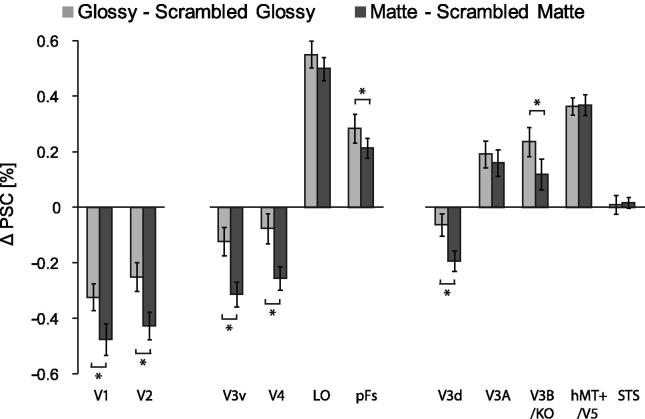
The **Δ**PSC for Glossy (light bars) and Matte (dark bars) conditions in all the ROIs. The **Δ**PSC in Glossy condition was calculated by [Glossy – Scrambled Glossy] PSCs and the **Δ**PSC in Matte condition was calculated by [Matte – Scrambled Matte] PSCs. The bars reflect mean **Δ**PSC across 15 participants with ±1 SEM. Asterisks represent significant difference between Glossy and Matte in ROIs (*p* < .01). The bars were arranges in three groups which represent the ROIs in early visual areas, ventral visual areas and dorsal visual areas respectively.

To complement our whole brain contrast analysis, we also examined the percent signal change (PSC) within independently identified regions of interest. To identify responses to global objects with consistent surface properties, we contrasted the glossy and matte stimuli against their scrambled controls by subtracting PSC in scrambled conditions from their intact counterparts for Glossy (light bars) and Matte (dark bars) conditions leading to **Δ**PSC (Fig. 5). We first tested whether activation differed for scrambled stimuli and their intact counterparts by testing if the **Δ**PSC deviated from zero. In early (V1, V2) and intermediate (V3d, V3v, V4) visual areas, we found stronger responses to the scrambled stimuli than their intact counterparts (single sample *t*-test, two-tailed, Bonferroni corrected, on **Δ**PSC averaged across Glossy and Matte conditions. V1: *t*_14_ = 8.5, *p* < .001; V2: *t*_14_ = 7.2, *p* < .001; V3d: *t*_14_ = 3.7, *p* < .022; V3v: *t*_14_ = 5.0, *p* < .001; V4: *t*_14_ = 3.8, *p* < .022), indicating that globally incoherent stimuli drive higher levels of activity. By contrast, in higher visual areas V3A, V3B/KO, hMT+/V5, LO and pFs we found stronger responses for intact versions of the stimuli (V3A: *t*_14_ = 4.0, *p* < .011; V3B/KO: *t*_14_ = 3.4, *p* < .044; hMT+/V5: *t*_14_ = 11.9, *p* < .001; LO: *t*_14_ = 12.7, *p* < .001; pFs: *t*_14_ = 5.9, *p* < .001). Response magnitudes in the STS were low, and not significantly different from zero (*t*_14_ = 0.5, *p* = .604).

**Supplementary Figure 1:**
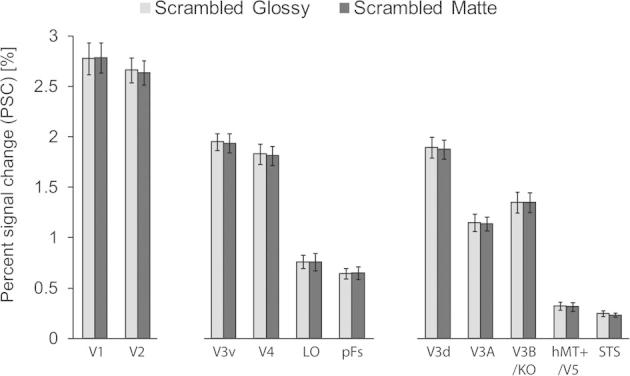
The PSC of Scrambled Glossy condition (light bar) and Scrambled Matte condition (dark bar). No significant difference was found between the two conditions in each ROI.

We then compared **Δ**PSC for Glossy (light bars) against Matte (dark bars) conditions (Fig. 5) in all the ROIs. A two-way repeated measures ANOVA showed a significant difference between Glossy and Matte conditions (*F*_1,14_ = 10.7, *p* = .006), an effect of ROI (*F*_10,140_ = 102.5, *p* < .001), and a significant interaction (*F*_10,140_ = 12.9, *p* < .001). Thereafter we tested for the differences between conditions in each ROI. Asterisks in Fig. 5 represent significant differences in activation between the two conditions (Tukey’s HSD post-hoc test at *p* < 0.01). We found that responses were significantly higher for objects with glossy than with matte surfaces in areas V3B/KO and pFs. Note that to compute **Δ**PSC we subtracted the activation in scrambled versions of the stimuli, so the glossy selectivity observed in V3B/KO and pFs is unlikely to be explained by low-level differences in the images of the objects. Moreover, we found no significant difference in the percent signal change (PSC) between Scrambled Glossy and Scrambled Matte conditions (see Supplementary Fig. 1), suggesting that the significant differences in **Δ**PSC between glossy and matte stimuli were mainly due to the PSC difference between Glossy and Matte conditions rather than between their scrambled counterparts. **Δ**PSC in early visual areas (V1, V2, V3v, V3d, V4) were also significant, however response modulation in these areas was higher for scrambled stimuli than for intact ones. Since the PSC in Scrambled Glossy and Scrambled Matte conditions were similar (see Supplementary Fig. 1), we can conclude that the difference is mainly due to intact conditions. It is possible that some neurons in these areas selectively respond to glossy object (Okazawa et al., 2012 and Wada et al., 2014 also found the importance of V1-V4 in gloss processing), however, unlike V3B/KO and pFs, these areas respond prevalently to scrambled images rather than intact ones. This suggests that these areas primarily deal with low-level image features and do not account for overall glossy appearance. As reviewed above, responses in STS were very low and not significantly different across conditions.

**Fig. 6:**
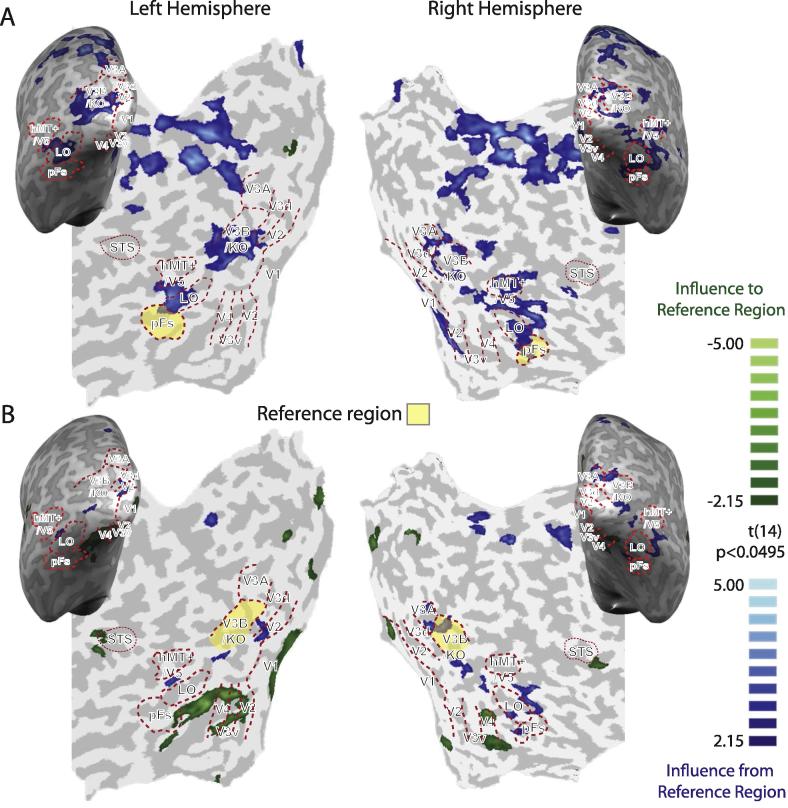
RFX GCMs with (A) pFs and (B) V3B/KO as reference regions (yellow areas). Blue areas received significant influence from the reference region and green areas sent significant influence to the reference region (*p* < .05 for *t*-test on GCMs). Note that since the group GCMs were averaged across participants and then presented on representative flat maps, individual ROI boundaries may not perfectly fit the group level.

The preceding analysis indicates two brain areas (pFs and V3B/KO) that appear to be important in processing information about gloss. To quantify how these areas communicate with other parts of the visual cortex, we used a random effects Granger causality mapping analysis (RFX GCM) to assess how these areas influence and depend on activity elsewhere. Fig. 6 shows the results using either pFs (Fig. 6A) or V3B/KO (Fig. 6B) as the reference region, respectively. Blue areas indicate brain areas that are significantly influenced by the reference region, while the green colour map identifies locations that have a significant influence on the reference region. We found that activity in pFs had a strong influence on both dorsal and ventral areas. This may reveal that gloss-related activity is used for the processes of object processing (in ventral cortex) in addition to affecting depth estimates (estimated in dorsal areas). By contrast, the estimated connectivity in V3B/KO was quite different. V3B/KO mainly received information from ventral areas rather than having influence on them, perhaps indicating that gloss information in V3B/KO is inherited from a primary locus in ventral areas. In addition, we observed that V3B/KO also received some information from an area near the STS. Although our other analyses did not suggest the involvement of the STS, this analysis appears consistent with the role of the STS in gloss indicated by electrophysiological recordings (Nishio et al., 2012). We should note that we could not determine whether the information flow captured by the Granger Causality Mapping is specific to gloss signals. Nevertheless, as the preceding conjunction analysis and PSC results showed the importance of pFs and V3B/KO in processing gloss, it is quite possible that the GCMs show different information flows between pFs and V3B/KO for gloss processing.

## 4 General discussion

The aim of this study was to localize the brain areas preferentially responding to glossy objects in the human brain. We did this by rendering glossy and matte versions of three-dimensional objects, and using scrambled images to control for low-level image cues. Our results point to a role for the posterior fusiform sulcus (pFs) and area V3B/KO in the processing of surface gloss: we found stronger responses to glossy objects than their matte counterparts, and this could not be explained by low-level stimulus differences. By assessing connectivity between brain areas while viewing glossy and matte stimuli, we observed that pFs exerted influence on ventral and dorsal brain areas, while V3B/KO was influenced by activity in midlevel ventral areas, which may indicate a difference between areas in their use of information from gloss as a cue to material (pFs) vs. object shape (V3B/KO).

Recent imaging studies in macaques suggest that glossy objects elicit more activation along the ventral visual pathway form V1 to IT cortex (Okazawa et al., 2012). We also found higher activation in the ventral stream, in particular in the pFs. Our results are reassuringly consistent with a very recent fMRI study that used a different image control approach (Wada et al., 2014). In particular, that study indicated the role of ventral areas and the combined areas V3A/B (which is very near to the V3B/KO that we identify). Since the ROI in our study were mapped using independent localisers before the experiment whereas Wada et al. considered only one area (V3A/B), our results pinpoint gloss-related activity more precisely, suggesting that the more lateral V3B/KO region is more important in gloss processing than V3A. The involvement of early visual areas (V1 to V4) is not clear. Although **Δ**PSC in earlier areas is significant due to higher activation for Glossy than for Matte objects (see Okazawa et al., 2012; Wada et al., 2014), however, unlike V3B/KO and pFs, response modulation in these early areas is higher for scrambled stimuli. This suggests that these areas primarily deal with low-level features such as the area which occupies visual field, discontinued borders and high spatial frequency information which is more in scrambled than in intact conditions. Note that some low-level features might be affected by our scrambling technique. For example, there are more highlight boundaries (line segments and edges) on Glossy objects and scrambling decreases the number of these segments and edges. Thus, the PSC difference in V1 to V4 might be caused by such low-level image properties rather than glossiness.

Previous human fMRI studies found the modulation of fMRI responses by different object materials perception in the fusiform gyrus (FG) and collateral sulcus (CoS) (Cant & Goodale, 2007; Cant et al., 2009; Cavina-Pratesi, Kentridge, Heywood, & Milner, 2010a,b; Hiramatsu et al., 2011). This work employed a wide variety of object materials (e.g., metal, wood, stone, glass) thus creating differences in surface gloss as well as differences in texture and colour. Here we focused on gloss, manipulating surface reflectance of untextured and homogeneously coloured objects. Despite this important difference between the studies, the surface-property-specific region (they denoted as CoS) they found is located very close to the area we denote as pFs based on a comparison of Talairach coordinates. Consistent with this, other work showed that a patient with colour and texture discrimination deficit could judge glossiness correctly, indicating that glossiness information does not exclusively depend on colour or texture processing (Kentridge et al., 2012). Taken together, this evidence suggests a dissociation between areas underlying material/texture from gloss. Nevertheless, the proximity of these areas may suggest a close interrelation and connection between material and gloss processing centres.

### 4.1 The role of V3B/KO in gloss processing

An important finding here is that the brain area V3B/KO seems to be involved in gloss processing. V3B/KO, located in dorsal visual stream, is well known to selectively respond to kinetic boundaries (Van Oostende, Sunaert, Van Hecke, Marchal, & Orban, 1997). It was also found to be involved in integrating different depth cues (Ban et al., 2012; Dövencioğlu et al., 2013; Murphy et al., 2013; Tyler, Likova, Kontsevich, & Wade, 2006). Our study, together with the recent results by Wada et al. (2014), indicate that the activity in V3B/KO is modulated by surface gloss, although previous work has not highlighted the involvement of this area in processing material information. One possibility is that V3B/KO does not actually processes gloss information *per se*. The causality mapping suggests quite a different pattern of causal relationships in V3B/KO than in pFs, with V3B/KO primarily being influenced by signals from elsewhere, while pFs influences responses in other areas. It is possible that the effect we found in V3B/KO was due to the effect of adding internal boundaries to the shapes corresponding to the locations with highlights. Alternatively, because specular highlights are known to influence the perception of 3D shape (Blake & Bülthoff, 1990; Fleming, Torralba, & Adelson, 2004; Muryy, Welchman, Blake, & Fleming, 2013), it is possible that differences in activity in V3B/KO for glossy vs. matte objects relate to differences in the estimated 3D shape. This appears consistent with the recent work that indicates that V3B/KO integrates different cues to 3D structure (Ban et al., 2012; Dövencioğlu et al., 2013; Murphy et al., 2013).

### 4.2 Human STS in gloss processing

The superior temporal sulcus (STS) of the macaque was found to show specific responses to glossy objects based on both fMRI (Okazawa et al., 2012) and single-unit recordings (Nishio et al., 2012). However, in our study we did not find strong evidence for the involvement of human STS in glossiness processing: changes in signals in this area were low, although the causality mapping did indicate some modulation of activity near the STS. It is possible that there are functional differences between human brain and monkey brain. For example, studies found functional differences between the two species in V3A and the intraparietal cortex for three-dimensional structure-from-motion (3D-SFM) processing (Orban, 2011; Vanduffel et al., 2002). It is also possible that the reasonably large voxel sizes used in our study limited our ability to detect responses to glossy stimuli in the human STS, and/or that the underlying population is spatially limited such that it did not survive the cluster threshold we applied.

### 4.3 The advantages of using mosaic spatial scrambling

In our study we chose to generate control stimuli using a scrambling technique applied to a visible grid. The presence of a grid reduces changes in low-level image properties due to scrambling (e.g., luminance, contrast, luminance histogram skew) while disrupting global properties of the shapes that are known to modulate the impression of gloss (Anderson & Kim, 2009; Kim et al., 2011; Marlow et al., 2011). The use of a superimposed grid over the stimuli was conceived to ensure that the amount of edge information in the stimuli was broadly similar between intact and scrambled conditions (Fig. 2). This expedient overcomes the large difference in spatial frequency content that would be produced by scrambling alone (Fig. 2D). Although there are slight differences in spatial frequency between intact objects and their scrambled counterparts (see Fig. 2D), scrambling had similar effects for Glossy and Matte conditions. Therefore differences in the spatial frequency spectra could not be the only cause for the pattern of results found. Furthermore, image statistics (luminance, contrast, skew and spatial frequency) did not differ substantially between Glossy and Matte conditions, ensuring that the results are not due to these properties as well. One could also argue that images with an overlaid grid could be amodally completed behind the occlusions. Such completion would be present for intact objects in both Glossy and Matte conditions. Therefore the completion-related activity would not bias the results. Similarly, even though scrambling clearly makes the stimuli occupy a larger portion of the visual field (Fig. 1), our analysis procedures makes it unlikely that such differences contributed to the findings we report in the study. This is because our conjunction analyses were not based only on [Glossy vs. Glossy Scrambled] and on [Matte vs. Matte Scrambled] comparisons, but also on the contrast [Glossy vs. Matte]. Overall, the results we presented cannot be explained by local edges, contrast, or configuration changes as these factors were the same for Glossy and Matte conditions.

We should also note that during our experiments our participants were not making active perceptual judgments of gloss. It is possible that activations would have been stronger had we asked for concurrent perceptual judgments. However, this would likely have introduced attention-based differences between the intact and scrambled conditions, which we deliberately sought to avoid using a task at the fixation point.

Finally, it is interesting to consider whether the areas we identify here would be involved in other aspects of gloss processing. As discussed in the Introduction, gloss perception can be modulated by several factors including low-level image cues (i.e. low-level image statistics), image configurations (such as the position and orientation of highlights), scene variables including light source direction (Marlow & Anderson, 2013; Marlow et al., 2012; Wijntjes & Pont, 2010), light source style (Dror, Willsky, & Adelson, 2004; Fleming, Dror, & Adelson, 2003; Marlow & Anderson, 2013; Olkkonen & Brainard, 2010; Te Pas, Pont, & van der Kooij, 2010) and background colour (Doerschner, Maloney, & Boyaci, 2010). Moreover, factors related to 3D structure from self motion and object motion (Doerschner et al., 2011; Sakano & Ando, 2010; Uehara et al., 2013; Wendt et al., 2010) and stereo viewing (Sakano & Ando, 2010; Sawabe, Yamamoto, Nakaguchi, Yamauchi, & Tsumura, 2010) can change perceived gloss. Finally, even non-visual sources such as haptic cues (Kerrigan, Adams, & Graf, 2010) and interactions with objects (Scheller Lichtenauer, Schuetz, & Zolliker, 2013) can lead to changes in surface appearance. It is an open challenge to understand whether these variables involve processing in pFs and V3B/KO, or whether additional areas are recruited.

## 5 Conclusion

This study reveals that V3B/KO and pFs are selectively active when processing images of glossy objects. This finding is consistent with other recent human fMRI studies and it suggests close but dissociated networks for gloss and material processing in the ventral stream. Our results point to a different role of V3B/KO and pFs, suggesting that V3B/KO may be tuned to processing highlight boundaries or 3D shape properties rather than to glossiness processing. Overall, our study highlights a small network in the fusiform sulcus that may be important in supporting our perception of surface gloss.

## References

[b0005] Anderson B.L., Kim J. (2009). Image statistics do not explain the perception of gloss and lightness. Journal of Vision.

[b0010] Anderson B.L., Kim J., Marlow P. (2011). At what level of representation is surface gloss computed?. Journal of Vision.

[b0015] Anderson B.L., Marlow P.J., Kim J. (2012). Disentangling 3D shape and perceived gloss. Journal of Vision.

[b0020] Ban H., Preston T.J., Meeson A., Welchman A.E. (2012). The integration of motion and disparity cues to depth in dorsal visual cortex. Nature Neuroscience.

[b9000] Beck J. (1972). Surface color perception.

[b0025] Blake A., Bülthoff H. (1990). Does the brain know the physics of specular reflection?. Nature.

[b0030] Brainard D.H. (1997). The psychophysics toolbox. Spatial Vision.

[b0035] Cant J.S., Arnott S.R., Goodale M.A. (2009). FMR-adaptation reveals separate processing regions for the perception of form and texture in the human ventral stream. Experimental Brain Research.

[b0040] Cant J.S., Goodale M.A. (2007). Attention to form or surface properties modulates different regions of human occipitotemporal cortex. Cerebral Cortex.

[b0045] Cavina-Pratesi C., Kentridge R.W., Heywood C.A., Milner A.D. (2010). Separate channels for processing form, texture, and color: Evidence from fMRI adaptation and visual object agnosia. Cerebral Cortex.

[b0050] Cavina-Pratesi C., Kentridge R.W., Heywood C.A., Milner A.D. (2010). Separate processing of texture and form in the ventral stream: Evidence from fMRI and visual agnosia. Cerebral Cortex.

[b0055] Doerschner K., Fleming Roland W., Yilmaz O., Schrater Paul R., Hartung B., Kersten D. (2011). Visual motion and the perception of surface material. Current Biology.

[b0060] Doerschner K., Maloney L.T., Boyaci H. (2010). Perceived glossiness in high dynamic range scenes. Journal of Vision.

[b0065] Dövencioğlu D., Ban H., Schofield A.J., Welchman A.E. (2013). Perceptual integration for qualitatively different 3-d cues in the human brain. Journal of Cognitive Neuroscience.

[b0070] Dror R.O., Willsky A.S., Adelson E.H. (2004). Statistical characterization of real-world illumination. Journal of Vision.

[b0075] Fleming R.W. (2012). Human perception: Visual heuristics in the perception of glossiness. Current Biology.

[b0080] Fleming R.W., Dror R.O., Adelson E.H. (2003). Real-world illumination and the perception of surface reflectance properties. Journal of Vision.

[b0085] Fleming R.W., Torralba A., Adelson E.H. (2004). Specular reflections and the perception of shape. Journal of Vision.

[b0090] Gegenfurtner K., Baumgartner E., Wiebel C. (2013). The perception of gloss in natural images. Journal of Vision.

[b0095] Hiramatsu C., Goda N., Komatsu H. (2011). Transformation from image-based to perceptual representation of materials along the human ventral visual pathway. NeuroImage.

[b0100] Junghöfer M., Schupp H.T., Stark R., Vaitl D. (2005). Neuroimaging of emotion: Empirical effects of proportional global signal scaling in fMRI data analysis. NeuroImage.

[b0105] Kentridge R.W., Thomson R., Heywood C.A. (2012). Glossiness perception can be mediated independently of cortical processing of colour or texture. Cortex.

[b0110] Kerrigan I.S., Adams W.J. (2013). Highlights, disparity, and perceived gloss with convex and concave surfaces. Journal of Vision.

[b0115] Kerrigan I.S., Adams W.J., Graf E.W. (2010). Does it feel shiny? Haptic cues affect perceived gloss. Journal of Vision.

[b0120] Kim J., Anderson B.L. (2010). Image statistics and the perception of surface gloss and lightness. Journal of Vision.

[b0125] Kim J., Marlow P., Anderson B.L. (2011). The perception of gloss depends on highlight congruence with surface shading. Journal of Vision.

[b0130] Kourtzi Z., Kanwisher N. (2000). Cortical regions involved in perceiving object shape. Journal of Neuroscience.

[b0135] Malach R., Reppas J.B., Benson R.R., Kwong K.K., Jiang H., Kennedy W.A. (1995). Object-related activity revealed by functional magnetic resonance imaging in human occipital cortex. Proceedings of the National Academy of Sciences.

[b0140] Marlow P., Anderson B.L. (2013). Generative constraints on image cues for perceived gloss. Journal of Vision.

[b0145] Marlow P., Kim J., Anderson B.L. (2011). The role of brightness and orientation congruence in the perception of surface gloss. Journal of Vision.

[b0150] Marlow P., Kim J., Anderson Barton L. (2012). The perception and misperception of specular surface reflectance. Current Biology.

[b0155] Motoyoshi I., Nishida S.y., Sharan L., Adelson E.H. (2007). Image statistics and the perception of surface qualities. Nature.

[b0160] Murphy A.P., Ban H., Welchman A.E. (2013). Integration of texture and disparity cues to surface slant in dorsal visual cortex. Journal of Neurophysiology.

[b0165] Muryy A.A., Fleming R.W., Welchman A.E. (2012). Binocular cues for glossiness. Journal of Vision.

[b0170] Muryy A.A., Welchman A.E., Blake A., Fleming R.W. (2013). Specular reflections and the estimation of shape from binocular disparity. Proceedings of the National Academy of Sciences.

[b0175] Nishida S.y., Motoyoshi I., Maruya K. (2011). Luminance-color interactions in surface gloss perception. Journal of Vision.

[b0180] Nishio A., Goda N., Komatsu H. (2012). Neural selectivity and representation of gloss in the monkey inferior temporal cortex. The Journal of Neuroscience.

[b0185] Nishio A., Shimokawa T., Goda N., Komatsu H. (2014). Perceptual gloss parameters are encoded by population responses in the monkey inferior temporal cortex. The Journal of Neuroscience.

[b0190] Norman J.F., Todd J.T., Orban G.A. (2004). Perception of three-dimensional shape from specular highlights, deformations of shading, and other types of visual information. Psychological Science.

[b0195] Okazawa G., Goda N., Komatsu H. (2012). Selective responses to specular surfaces in the macaque visual cortex revealed by fMRI. NeuroImage.

[b0200] Okazawa G., Koida K., Komatsu H. (2011). Categorical properties of the color term “GOLD”. Journal of Vision.

[b0205] Olkkonen M., Brainard D.H. (2010). Perceived glossiness and lightness under real-world illumination. Journal of Vision.

[b0210] Orban G.A. (2011). The extraction of 3D shape in the visual system of human and nonhuman primates. Annual Review of Neuroscience.

[b0215] Orban G.A., Fize D., Peuskens H., Denys K., Nelissen K., Sunaert S. (2003). Similarities and differences in motion processing between the human and macaque brain: evidence from fMRI. Neuropsychologia.

[b0220] Pelli D.G. (1997). The VideoToolbox software for visual psychophysics: Transforming numbers into movies. Spatial Vision.

[b0225] Preston T.J., Li S., Kourtzi Z., Welchman A.E. (2008). Multivoxel pattern selectivity for perceptually relevant binocular disparities in the human brain. The Journal of Neuroscience.

[b0230] Roebroeck A., Formisano E., Goebel R. (2005). Mapping directed influence over the brain using Granger causality and fMRI. NeuroImage.

[b0235] Sakano Y., Ando H. (2010). Effects of head motion and stereo viewing on perceived glossiness. Journal of Vision.

[b0240] Sawabe M., Yamamoto S., Nakaguchi T., Yamauchi Y., Tsumura N. (2010). A study for gloss perception on stereo display using magnitude estimation method. Journal of Vision.

[b0245] Scheller Lichtenauer M., Schuetz P., Zolliker P. (2013). Interaction improves perception of gloss. Journal of Vision.

[b0250] Sunaert S., van Hecke P., Marchal G., Orban G.A. (1999). Motion-responsive regions of the human brain. Experimental Brain Research.

[b0255] Te Pas S.F., Pont S.C., van der Kooij K. (2010). Both the complexity of illumination and the presence of surrounding objects influence the perception of gloss. Journal of Vision.

[b0260] Tyler C.W., Likova L.T., Kontsevich L.L., Wade A.R. (2006). The specificity of cortical region KO to depth structure. NeuroImage.

[b0265] Uehara T., Tani Y., Nagai T., Koida K., Nakauchi S., Kitazaki M. (2013). Effects of retinal-image motion of specular highlights induced by object motion and manual control on glossiness perception. Journal of Vision.

[b0270] Van Oostende S., Sunaert S., Van Hecke P., Marchal G., Orban G.A. (1997). The kinetic occipital (KO) region in man: An fMRI study. Cerebral Cortex.

[b0275] Vanduffel W., Fize D., Peuskens H., Denys K., Sunaert S., Todd J.T. (2002). Extracting 3D from Motion: Differences in Human and Monkey Intraparietal Cortex. Science.

[b0280] Wada A., Sakano Y., Ando H. (2014). Human cortical areas involved in perception of surface glossiness. NeuroImage.

[b0285] Wendt G., Faul F., Ekroll V., Mausfeld R. (2010). Disparity, motion, and color information improve gloss constancy performance. Journal of Vision.

[b0290] Wendt G., Faul F., Mausfeld R. (2008). Highlight disparity contributes to the authenticity and strength of perceived glossiness. Journal of Vision.

[b0295] Wijntjes M.W.A., Pont S.C. (2010). Illusory gloss on Lambertian surfaces. Journal of Vision.

